# Numerical Simulation of the Performance of Sb_2_Se_3_ Solar Cell via Optimizing the Optoelectronic Properties Based SCAPS-1D

**DOI:** 10.3390/ma15186272

**Published:** 2022-09-09

**Authors:** Shahbaz Abbas, Saraswati Bajgai, Shahariar Chowdhury, Asmaa Soheil Najm, Mohammad Shah Jamal, Kuaanan Techato, Sittiporn Channumsin, Suwat Sreesawet, Manun Channumsin, Amel Laref, Kazi Sajedur Rahman, Araa Mebdir Holi

**Affiliations:** 1 Faculty of Environmental Management, Prince of Songkla University, Hat Yai, Songkhla 90110, Thailand; 2 Chemical Engineering Department, University of Technology, Baghdad 10066, Iraq; 3 Institute of Fuel Research and Development (IFRD), Bangladesh Council of Scientific and Industrial Research(BCSIR), Dhaka 1205, Bangladesh; 4 Space Technology Research Centre, Geo-Informatics and Space Technology Development Agency (GISTDA), Chonburi 20230, Thailand; 5 Faculty of Veterinary Medicine, Rajamangala University of Technology Tawan-Ok, Chonburi 20110, Thailand; 6 Physics Department, College of Science, King Saud University, Riyadh 11451, Saudi Arabia; 7 Solar Energy Research Institute (SERI), Universiti Kebangsaan Malaysia, Bangi 43600, Selangor, Malaysia; 8 Department of Physics, College of Education, University of Al-Qadisiyah, Al-Qadisiyah, Al-Diwaniyah 58002, Iraq

**Keywords:** solar cells, antimony triselenide, career concentration, bandgap, SCAPS-1D

## Abstract

Antimony trisulfide (Sb_2_Se_3_), a non-toxic and accessible substance, has possibilities as a material for use in solar cells. The current study numerically analyses Sb_2_Se_3_ solar cells through the program Solar Cell Capacitance Simulator (SCAPS). A detailed simulation and analysis of the influence of the Sb_2_Se_3_ layer’s thickness, defect density, band gap, energy level, and carrier concentration on the devices’ performance are carried out. The results indicate that a good device performance is guaranteed with the following values in the Sb_2_Se_3_ layer: an 800 optimal thickness for the Sb_2_Se_3_ absorber; less than 10^15^ cm^−3^ for the absorber defect density; a 1.2 eV optimum band gap; a 0.1 eV energy level (above the valence band); and a 10^14^ cm^−3^ carrier concentration. The highest efficiency of 30% can be attained following optimization of diverse parameters. The simulation outcomes offer beneficial insights and directions for designing and engineering Sb_2_Se_3_ solar cells.

## 1. Introduction

Solar energy can be harnessed to fulfil expanding energy requirements. Increasing solar energy utilization also demands innovative photovoltaic (PV) technologies with cheap mass production costs and an excellent power conversion efficiency (PCE) [1]. Thin-film photovoltaic (TFPV) technologies have drawn huge research focus due to the benefits of lesser material consumption, greater power production, and scalable flexibility [2,3,4]. For the different types of thin-film solar cells, notable successes have been attained in the representative cadmium telluride (CdTe) [5], copper indium gallium selenide (CIGS) [6,7], and perovskites [8,9]. The paucity of indium (In) and toxicity of cadmium (Cd) restrict their long-term use. Numerous low toxic and earth-abundant photoabsorber materials, such as CZTS(e) SnS Cu_2_O, CuSbSe_2_, and Sb_2_Se_3_, have been explored for PV applications [10,11]. Among those emerged as potential candidates for next generation light-harvesting materials of the chalcogenides, antimony selenide (Sb_2_Se_3_) has high absorption coefficients (>10^5^ cm^−1^), a six suitable band-gap energy of 1.1–1.2 eV, and a 6–8 earth-abundant low-toxic constituent [12,13]. It is an innocuous and economical material having a band gap of ~1.2 eV [14]. Sb_2_Se_3_ has drawn considerable interest in the past few years due to its sound photo-conducting attributes and superior thermoelectric power [15], which aid uses in optical and thermoelectric cooling tools. Besides these uses, Sb_2_Se_3_ has diverse uses in photodetectors, solar cells, batteries, and memory gadgets [16]. All these uses are strongly reliant on the electrical, optical, microstructural, and other attributes of Sb_2_Se_3_. Material synthesis and deposition too play a crucial part in attaining an improved quality of materials. In the past decade, Sb_2_Se_3_ solar cells have been broadly studied, uncovering significant advancement with PCEs of 3.21%, 7.6%, and 9.2% [2]. 

Several aspects were ascribed to this enhanced PCE, such as improved absorber growth procedures (such as vapour transport deposition, close space sublimation, and hydrothermal), buffer layer optimisation (CdTe, ZnO, and TiO_2_) and innovative architectural designs of the device [17]. There was an improvement in the device performance despite the Sb_2_Se_3_ cells’ display of photovoltage in a low value. Several studies by [18] noted that based on the numerical simulations, after the suppression of the negative band bending, additional holes were removed from the rear contact. It was pointed out that, compared to other solar cells, the antimony chalcogenide (such as Sb_2_Se_3_) solar cells called for a better hole transport layer (HTL). In addition, Chen et al. (2017) [19] reported that the Sb_2_Se_3_ efficiency was improved by the inorganic PbS colloidal quantum dot film from 5.42 to 6.50%. Several researchers utilised an inorganic copper(I) thiocyanate (CuSCN)-HTL to increase the Sb_2_Se_3_ cells’ PCE by 7.5%. The researcher used the following common HTL in different solar cells: 2,2,7,7-Tetrakis (N, N-di-p-methoxyphenylamine)- 9,9-spirobi-fluorene (Spiro-OMeTAD). It was likewise utilised in the Sb_2_ (S, Se)_3_ solar cells that have a <10% PCE value [20,21]. Nonetheless, using organic HTLs for large-scale production is not advisable because of their erratic device performance and expensive nature. Therefore, it is imperative to design a new, stable, cost-effective, and non-toxic material in order to manufacture efficient photovoltaic (PV) cells. 

Applying this technique is fairly simple and is broadly employed for examining the HTLs’ efficiency in perovskite devices. Moreover, PEDOT: PSS is regarded to be cost-friendly and offers high-quality results, making it an optimum choice as an HTL for the development of PSC. Even though the use of PEDOT: PSS as an HTL for PSCs is broad and effective, it shows poor performance as well as stability versus other HTLs due to its highly doped characteristic. This results in numerous issues, such as acute interfacial recombination. Thus, PEDOT: PSS conductivity was altered and enhanced by the researchers via additional p-doping in order to match the energetics as well as enhance the device performance. As an ETL, TiO_2_ is employed and generally annealed at >450 °C for synthesising TiO_2_ crystals. The evaluation of some of the metal oxides, which were treated under low temperatures, was done as ETLs in the PSCs in order to avoid annealing at high temperatures, such as SnO_2_ [22], In_2_O_3_ [3], WO_3_ [23], amorphous-TiO_x_ [24], Zn_2_SnO_4_ [25], La-doped BaSnO_3_ [21], and ZnO [26]. From these ETLs, ZnO could be considered as a potential ETL as an ultrahigh electron mobility (205–300 cm^−2^ /Vs) was observed [26]. Benami et al. (2022), found that, compared to TiO_2_, the use of ZnO as an electron transport material increases cell efficiency [27]. As in a perovskite solar cell, the lower edge of the conduction band enhances the transit of photogenerated electrons. 

Various researchers have used different techniques to enhance the ZnO semiconductor characteristics, and subsequently the PSCs’ photovoltaic performance via doping and designing ZnO by employing other metal oxides or elements [28]. Even though these experiments were conducted, it was challenging for the researchers to enhance ZnO-based PSCs. ZnS was found to be similar with that of a ZnO wide bandgap semiconductor and it also showed comparable physical characteristics. With regards to quantum-dot-sensitised solar cells, ZnS showed outstanding electron mobility and was also seen to function similar to ETL and the interfacial passivation layer [28]. ZnS is associated with a low conduction band minimum (CBM) versus ZnO, making it a better match for MAPbI_3_-LUMO [28]. However, PSCs’ photovoltaic performance still needs more improvement by employing ZnS or ZnO in order to facilitate the transportation of electrons to ZnO from MAPbI_3_ [29], In the past, a poor photovoltaic performance was displayed by the ZnS-based PSCs. The ZnO/ZnS nanoparticle structure can be employed to considerably decrease the optical band gap while simultaneously maintaining the required optical absorption value. As per the theory, the PSCs’ open-circuit voltage (*V_oc_*) follows the energy difference between the valence band maximum (VBM) and CBM (ELT) [30]. Thus, the *V_oc_* value can be increased by adding ZnS to the ZnO-based PSCs [31]. Adding ZnS to the surfaces of ZnO behaves like an energy barrier that blocks the recombination of charges between MAPbI_3_ and ZnO [32]. However, this could also result in increasing ZnO-based PSCs’ short-circuit current.

This study concentrated on numerical simulation for demonstrating the link of carrier concentration, band gap, and energy level of the absorber layer on the antimony trisulfide solar cell’s PCE. To consider different permutations about absorber thickness and identification of the quantum impact concerning solar cell parameters to increase simulation outcomes’ applicability. 

## 2. Materials and Methods

**Figure 1:**
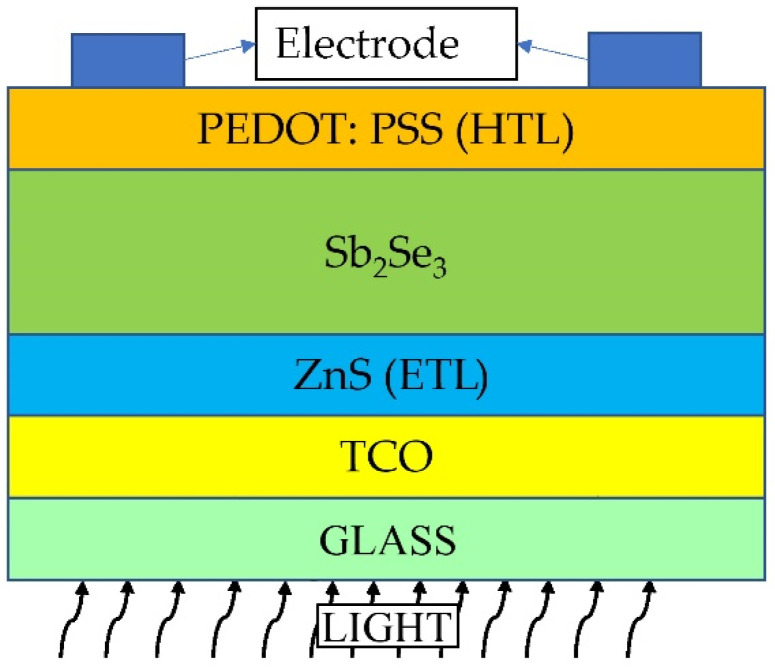
Structure of the Sb_2_Se_3_ solar cell device.

Here, the Sb_2_Se_3_ absorber-based TFSC was modelled and simulated by the researchers making use of the SCAPS-1D program, which can be defined as a 1D solar cell device simulator previously developed at the University of Gent [28]. Simulation of SCAPS-1D aided in solving the Poisson’s, drift-diffusion, and carrier continuity equations, which are the fundamental equations employed for designing a semiconductor device [33]. By employing the SCAPS-1D simulator, the researchers studied PV devices’ various properties, such as current–voltage (I–V), capacitance–voltage (C–V), and capacitance–frequency (C–F), as well as their EQE and recombination profiles. The results showed that the device performance is impacted by factors such as doping concentration, electron affinity, interface defect density, cell thickness, operating temperature, bulk defect density, resistances, and quantum efficiency metal work function. All simulations were conducted by maintaining the following conditions: illumination of 100 mWcm^−2^, temperature = 300 K, and AM1.5 G light spectrum. The ZnS/Sb_2_Se_3_/PEDOT: PSS heterojunction device structure is shown in Figure 1, and the energy level pertaining to the TFSC structure in Figure 1. 

As per the structure, the ultrathin p-type PEDOT: PSS as HTL connected the p-type Sb_2_Se_3_ absorber as well as rear metal contact. In the device, fluorine-doped tin oxide (F: SnO_2_) and the n-type ZnS were included in the TCO layer and ETL. Moreover, in the TFSCs, aluminium (Al) was employed to construct the front and rear metal contacts [34]. Table 1 shows the physical parameters that were considered in the simulation. The values of material parameters employed in the numerical computation were taken from the literature. The travelling speed of holes and electrons was at 1 × 10^7^ cm s^−1^ [35]. This study employed the SCAPS-1D software (3.3.10) to demonstrate the absorption coefficients pertaining to the ZnS and PEDOT:PSS as HTL materials, while the absorption coefficient values pertaining to the PEDOT:PSS (HTL) materials and Sb_2_Se_3_ absorber were taken based on the literature [35]. As previously noted, comparable defects were seen while the researchers utilized a Gaussian distribution function. Setting of the energy levels pertaining to the defects was done to mid-bandgap. They also involved absorber thickness/defect densities and ETL/absorber in order to determine the heterojunction TFSC interface carrier recombination. Table 1 shows all parameters pertaining to the interface faults. 

SCAPS–1D was employed for solving Poisson’s equation pertaining to electrons and holes (Equation (1)), as given below [45]:

(1) $$\frac{d^{2}\Psi }{dx^{2}}=\frac{e}{\in_{0}\in_{r}}\left[P\left(x\right)-n\left(x\right)+N_{D}-N_{A}+\rho_{P}-\rho_{n}\right]$$

Here, x = electrostatic potential; Ψ = elementary charge; $\in_{r}\;$= relative permittivity; $\in_{0}$ = vacuum permittivity; *P* = concentration of holes and electrons; *N_D_*, *N_A_* refer to the donor and acceptor charges; and $\rho_{p}$ and $\rho_{n}$ refer to the concentration of holes and electrons, respectively.

With regards to the theoretical perspective, the carrier lifetime τ has been defined as the period in which the charge carrier can move freely, allowing it to contribute to the electric conduction. Based on a uniform simulation of the Sb_2_Se_3_, development of the *G* (generation rate) electron-hole pairs is done. Equations (2) and (3) define the developed electron and hole densities with regards to *E_c_* and *E_v_*, as represented below [46]:

(2) $$\Delta n=G\tau_{n}$$

(3) $$\Delta p=G\tau_{p}$$

Should the above-mentioned carriers be trapped in perovskite as well as thermally recited, then the spent time on these traps was not considered in the $\tau_{n}$ and $\tau_{p}$. With regards to steady-state, it was seen that the rate of generation pertaining to the PSC was equal with the rate of trapping, as presented in Equations (3) and (4) below [46]:

(4) $$\tau_{n}=\frac{1}{\sigma_{p}.v_{th}.\left(N_{t}-N_{r}\right)}$$

(5) $$\tau_{\rho }=\frac{1}{\sigma_{p}.v_{th}.n_{r}}\;$$

wherein, $N_{t},\;n_{r},\;\sigma_{n},\;\mathrm{and}\;v_{th}\;$refer to the total (i.e., occupied and unoccupied), occupied defects, hole capture cross-section, and the thermal velocity, respectively. As per Equations (4) and (5), the increase in defect density resulted in a reduction in carrier lifetime as well as a short carrier diffusion length (*L*) [47]. This *L* value accounted for the perovskite quality. It was seen that when there was a higher *L* value versus Sb_2_Se_3_ thickness, the performance of the device can be enhanced. The following equations were constructed based on recombination current (*J_o_*), *L* and *V_oc_*:

(6) $$J_{o}\approx q\frac{Dn_{i}^{2}}{LN}$$

(7) $$V_{oc}=\frac{KT}{q}1n\left(\frac{J_{sc}}{J_{o}}+1\right)$$

whereby, *q*, *Dn*, *LN*, *K*, and *T* are electric charge of electron, diffusion constant, longer carrier diffusion length, Boltzmann constant, and temperature, respectively. As per Equations (6) and (7), a decrease in the defect density resulted in a reduction of recombination current as well as an increase in *V_oc_*, which was found to be comparable to the study results. The internal quantum efficiency (IQE) and *J_sc_* were observed to be directly proportional to each another. The IQE can depend on the minority carrier diffusion lengths, if the researchers consider the PSC to be a shallow junction solar cell along with a long minority carrier lifetime, as represented in Equation (8).

(8) $$\mathrm{IQE}=1-\alpha t-\frac{B}{\alpha L^{2}}$$

wherein *α*, *t*, and *B* indicated the spectral absorption coefficient, distance in the perovskite material, and Sb_2_Se_3_ thickness, respectively [48]. We employed the Shockley–Read–Hall (SRH) recombination for describing the recombination components that impacted the defect density in the PSC, as represented below

(9) $${ℜ}^{\mathrm{SRH}}=\frac{\vartheta \sigma_{n}\sigma_{p}N_{T}\left[np-n_{i}^{2}\right]}{\sigma_{p}\left[p+p_{1}\right]+\sigma_{n}\left[n+n_{1}\right]}$$

where *ϑ* = electron thermal velocity, *N_T_* = no. of defects per volume, *n_i_* + intrinsic number density, *n* and *p* = concentration of electrons and holes at equilibrium; and *n*_1_ and *p*_1_ = conc. of electrons and holes in the trap defect and valence band, respectively. 

## 3. Results and Discussions

### 3.1. Effect of Absorber Layer Thickness with QE

**Figure 2:**
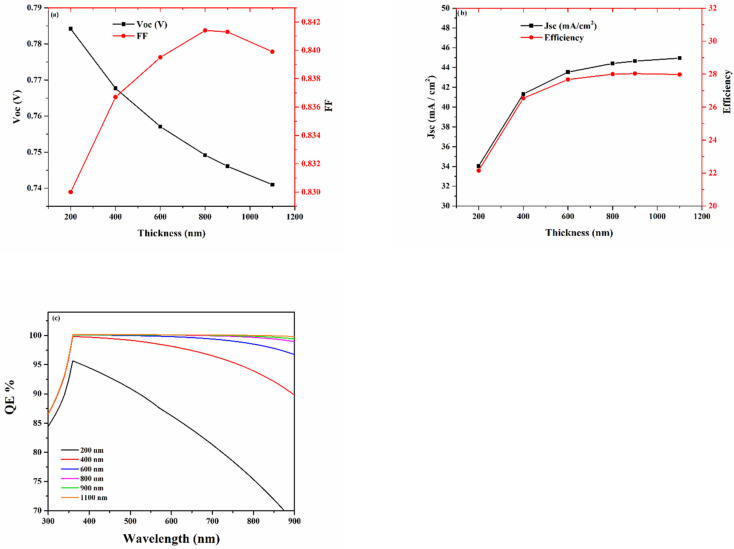
Absorber layer thickness with (**a**) *V_oc_* and FF, (**b**) *J_sc_* and efficiency, and (**c**) quantum efficiency.

**Figure 3:**
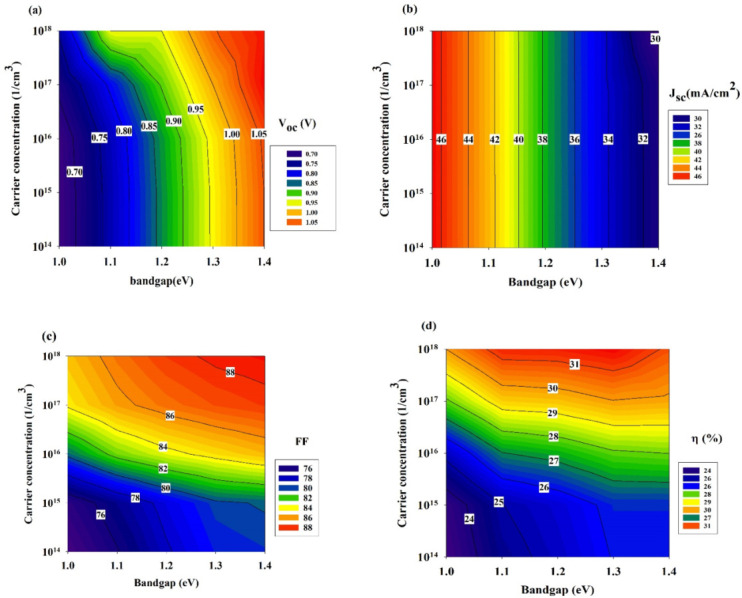
Photovoltaic performance parameters at various carrier concentration as: (**a**) *V_oc_*, (**b**) *J_sc_*, (**c**) FF, and (**d**) efficiency.

Figure 2 shows the (a) current density, (b) voltage characteristic, and (c) quantum efficiency (QE) produced with Table 1 preliminary parameters. In Figure 2a, *V_oc_* exhibited a decrease to a 200–800 nm thickness, which is the optimum value; it did not show any change after that. This indicates that in thicker films, the charge recombination increases. FF initially increases when the thickness reaches 200–800 nm and then it decreases from 84.14% to 83.99% when the thickness is further increased from 900 to 1100 nm; this is attributable to the incremental series resistance. Figure 2b illustrates the following simulation results: the *J_sc_* short circuit current increased when the absorber thickness is increased; when the thickness peaks to 1100 nm, the *J_sc_* reaches 44.95 mA/cm^2^, saturating to a plateau. If the FF and *V_oc_* trends with a varied Sb_2_Se_3_ thickness are taken into consideration, 800 nm, the optimal absorber thickness, is achieved, as can be seen in Figure 3b, which corresponds to a 28% PCE. This is because when the absorber thickness is too low, it does not benefit from the full absorption of light, therefore resulting in a low PCE. On the other hand, an absorber layer that is too thick will cause the photo-generated carriers to take a longer transfer route; this leads to higher recombination. The following simulations show that in the dark, when the thickness of the Sb_2_Se_3_ is set to the 800 nm optimum value, the device displays a current density value at the extreme minimum; this can be ascribed to the minority carrier. The reflection of the interface with each layer as well as that of the series and shunt resistance are not taken into account during the simulation. Figure 2c reveals the changes in the QE: it completely encompasses the visible spectrum while exhibiting a 300 to 900 nm range in high spectral response, which comes with a significant absorption onset rising up to 500 nm. In addition, the subsequent parasitic absorption of the absorber is responsible for the shorter wavelength region.

### 3.2. Carrier Concentration vs. Bandgap

This section exhibits the efforts undertaken towards clarifying the various absorber layers’ trend influence: through an analysis of the crucial photovoltaic performance characteristics, as these ultimately govern the efficiency of the solar cell conversion. Figure 3 demonstrates that *J_sc_* is reduced as the bandgap is increased, while *V_oc_*, a direct function of the bandgap, is also increased. A higher bandgap leads to a *V_oc_* increase and a rate of radiative recombination decrease [49]. Conversely, the *J_sc_* parameter changes significantly, as indicated in Figure 3. These changes are generally within the 30 to 46 (mA/cm^2^) range. If the concentration is relatively low, less carrier collection occurs at the front contact, and this leads to a reduced *J_sc_* [50]. Despite that, it is possible to maintain a high *J_sc_* value using a buffer layer that has a higher concentration of carriers. In our scenario, however, no noticeable improvement is observed even with a higher concentration of carriers. The optimal band alignment at the buffer/absorber interface (i.e., increased carrier concentration and bandgap) may explain the said occurrence. Such an alignment is crucial in efficient solar cells towards increasing the *J_sc_* [51]. This may be due to the fact that the carriers may have enhanced diffusivity, because of their increased mobility. 

Alternatively, because of a widening bandgap and constant carrier concentration, FF decreases. PCE showed improvement at the absorber layer’s higher bandgap in spite of the higher carrier concentration’s influence and regardless of the fact that PCE is considered to be the function of FF, *V_oc_*, and *J_sc_*. Our findings demonstrate that the optimal performance began at a 1.2 eV bandgap and a 1 × 10^16^ (1/cm^3^) defect density.

We assert that if the optical qualities of the connected absorber layer are not properly defined and adjusted, it may result in erroneous predictions regarding the real potential of the examined p-absorber material [52]. For example, consider that Sb_2_Sb_3_, having a 1.04 eV bandgap, is being examined, because of the material’s polycrystalline nature and the process of non-optimised deposition, it is quite possible that the thin film of the absorber will already have a bulk defect density during the early phases of the design process. In this situation, the assembled absorber layer exhibits a low carrier concentration, and it is likely that the associated device will have an efficacy that is below 24% (see Figure 3d). Nonetheless, the device’s efficiency could be boosted to an even greater level using an absorber layer having a greater carrier concentration.

### 3.3. Defect Density vs. Energy Level

**Figure 4:**
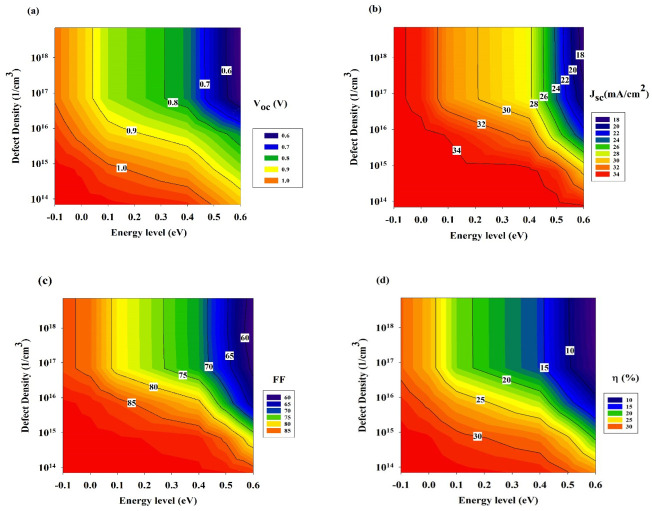
Photovoltaic performance parameters at various defect density as: (**a**) *V_oc_*, (**b**) *J_sc_*, (**c**) FF, and (**d**) efficiency.

Another crucial characteristic that can dramatically affect device performance is the absorber layer’s defect density. The greater concentrations of deficiencies in the absorber layer produce a greater rate of recombination due to the growth of pinholes, and also a higher rate of the film’s degradation, a lower strength, and a decrease in the device’s overall performance [49]. On the other hand, when there is low defect density, the length of the carrier diffusion is enhanced, and the process of the recombination process is rejected, both of which cause an improved PV performance. Figure 4a–d shows the effect that the defect density of the absorber layer has on the most significant parameters; the modelling was accomplished by modifying the defect density from 1 × 10^14^ cm^−3^ to 1 × 10^18^ cm^−3^.

As one can see, cell performance worsens as the absorber layer’s overall defect density increases. The defect density has a substantial effect on FF, *V_oc_*, and *J_sc_* since it is an essential parameter affecting the cell’s performance. It suggests that the radiative recombination had a minor effect on electron-hole pair formation and passage in solar cells, and the impact of modifying the *J_sc_* defect states was insignificant until it reached around 1 × 10^17^ cm^−3^. It is also fairly clear that the significant alterations in *V_oc_*, ranging from 0.6 to 1.0 V, are due to the existent defect energy level. These changes can be observed quite clearly. Conversely, the effect of radiative recombination on FF was noticeably more distinct. The cause for the FF decline that was seen in association with an increase in the radiative recombination rate was that electron-hole pairs were carried through the cell less effectively, causing an increase in the energy quantity that was lost to recombination [50]. For instance, at a 0.55 eV energy level, the FF is as low as ~65% with a defect density of 1 × 10^18^ cm^−3^, and it increases to 85% when the defect density is below 1 × 10^14^ cm^−3^. Moreover, as the defect density increases from 1 × 10^14^ cm^−3^ to 1 × 10^18^ cm^−3^, the efficiency declines drastically, the decline being between 10% and 30% (as shown in Figure 4d). It can be concluded that the defect density has a direct impact on the efficiency since as the number of defects goes higher, the charge carrier diffusion length decreases and it causes an introduction of more recombination carriers to the absorber layer [51]. The presence of a greater defect density leads to a higher recombination rate, as a result of which, the cell performance is affected. The optimal efficiency of the cell of 30% was attained by fine-tuning the *V_oc_* at 1.0 eV, the *J_sc_* at 34 mA/cm^2^, and FF at 85%, and the defect density at 1 × 10^14^ cm^−3^. 

## 4. Conclusions

In this research, the development of the antimony triselenide solar cells was optimised by using the SCAPS simulator for the p-i-n arrangement. They patterned the primary SC on the basis of the TCO/ZnS/Sb_2_Se_3_/PEDOT: PSS arrangement. For an optimised solar cell design, we used some parameters, such as the absorber layer thickness (ranging from 200 to 1100 nm), the carrier concentration (from 10^14^ to 10^18^ cm^−3^), and the defect density (which ranged from 10^14^ to 10^18^ cm^−^^3^) with an energy level (above valence band) ranging from −0.1 to 0.6 eV. The simulations exhibited a PCE of >30%, at the *V_oc_* of 1.0 eV, *J_sc_* of 34 mA/cm^2^, and FF of 85%. Moreover, it was noticed that the efficacy of the antimony triselenide solar cells got higher as the thickness of the absorber layer increased. Furthermore, the results indicated that a bandgap of 1.2 eV nm and a carrier concentration of 10^14^ cm^−3^ was satisfactory. This research simulates the thickness of the absorber layer and the quantum efficiency impact on the performance of the device. The absorber layer thickness had been varied (ranging from 200 to 1100 nm) along with the defect densities (ranging from 10^14^ to 10^18^ cm^−3^) and the energy level (ranging from −0.1 to 0.6 eV). The outcomes indicated that the device performance was highly susceptible to the defect densities; nonetheless, it exhibited a similar pattern in case of the absorber layer thickness. When the thickness of the absorber layer was increased from 300 nm to 900 nm, it considerably increased the PCE. Conversely, when its value was increased from 1000 nm to 1100 nm, it exhibited a slight drop in the PCE. The investigators concluded that the design of the device and the optimisation of the Sb_2_Se_3_ solar cells could significantly benefit from their experimental outcomes.

## Figures and Tables

**Table 1:** List of various simulation parameters involved in the perovskite planar structure.

Properties	ZnS (ETL)	Sb_2_Se_3_	PEDOT: PSS (HTL)	References
Thickness (nm)	70	variable	40	[29,36]
Bandgap, *E_g_* (eV)	3.5	variable	2.2	[29,35,37]
Electron Affinity, $x_{e}$ (eV)	4.5	4.04	2.9	[38,39,40]
Dielectric permittivity, $\in_{r}$ (relative)	10	18	3	[38,41,42]
CB effective density of states, *N_C_* (cm^−3^)	1.5 × 10^18^	2.2 × 10^18^	2.2 × 10^15^	[39,42,43]
VB effective density of states, *N_V_* (cm^−3^)	1.8 × 10^18^	1.8 × 10^19^	1.8 × 10^18^	[39,42,43]
Electron thermal velocity (cm/s)	1 × 10^7^	1 × 10^7^	1 × 10^7^	[34,37,41]
Hole thermal velocity	1 × 10^7^	1 × 10^7^	1 × 10^7^	[34,37,41]
Electron mobility (cm²/Vs)	50	15	10	[39,42,44]
Hole mobility (cm²/Vs)	20	5.1	10	[39,42,44]
Shallow uniform donor density, *N_D_* (cm^−3^)	1 × 10^22^	0	0	[39]
Shallow uniform acceptor density, *N_A_* (cm^−3^)	0	variable	3.17 × 10^14^	[34,39]
Defect density *N_t_* (cm^−3^)	1 ×10^14^	variable	1 × 10^16^	[34,39]
Energy level	0.6	variable	0.6	[25]

## Data Availability

Not Applicable.
